# Population Structure and Mating Type Distribution of *Cercospora sojina* from Soybeans in Indiana, United States

**DOI:** 10.3390/jof10110802

**Published:** 2024-11-19

**Authors:** Guohong Cai, Leandro Lopes da Silva, Natalia Piñeros-Guerrero, Darcy E. P. Telenko

**Affiliations:** 1 Crop Production and Pest Control Research Unit, Agricultural Research Service, United States Department of Agriculture, West Lafayette, IN 47907, USA; silva114@purdue.edu; 2 Botany and Plant Pathology Department, Purdue University, West Lafayette, IN 47907, USA; npierosg@purdue.edu (N.P.-G.); dtelenko@purdue.edu (D.E.P.T.)

**Keywords:** soybean, frogeye leaf spot, *Cercospora sojina*, single nucleotide polymorphism (SNP) markers, mating type, population structure

## Abstract

Frogeye leaf spot on soybeans is traditionally considered as a southern disease in the United States but its impact in North Central USA has been rising in recent years. In this study, we investigated the population structure and mating type distribution in the *C. sojina* population from Indiana, USA. Based on 27 single nucleotide polymorphism markers, 49 multi-locus genotypes (MLGs) were identified in 234 isolates collected from 29 counties in Indiana in 2020. Bayesian analysis grouped the 49 MLGs into three clusters. This grouping was supported by principal coordinate analysis and, in large part, by the unweighted pair group method with arithmetic mean and minimal spanning tree. Only one mating-type idiomorph was found in each isolate and in each MLG. The *MAT1-1* idiomorph was found in 22 MLGs and the *MAT1-2* idiomorph was found in 27 MLGs. Based on clone-corrected data, the distribution of mating-type idiomorphs did not deviate significantly from 1:1 ratio in Indiana as a whole and in 22 out of 24 counties where two or more MLGs were found. Thirty MLGs contained QoI-resistant isolates and 22 MLGs contained QoI-sensitive isolates, with three MLGs containing both types of isolates. MLG1, the most common MLG with 90 isolates, contained mostly QoI-resistant isolates. Interestingly, MLG1 was also the dominant genotype in the Tennessee population collected in 2015, suggesting that MLG1 has been a dominant genotype in a wider region for many years. Based on the standard index of association ($\bar{r}$_d_), the Indiana population as a whole was in significant linkage disequilibrium. However, in five out of 16 counties where three or more MLGs were found, the null hypothesis of linkage equilibrium was not rejected. Tests of linkage disequilibrium between locus pairs showed that 33.3% of locus pairs on the same contigs were in significant disequilibrium and 17.7% of locus pairs on different contigs were in significant disequilibrium. The possibility of a cryptic sexual stage was discussed.

## 1. Introduction

Frogeye leaf spot (FLS), caused by the fungal pathogen *Cercospora sojina* Hara, is a common disease of soybeans (*Glycine max* Merr.) found in most soybean-producing countries (reviewed in [1,2]). It was first reported in Japan in 1915 [3] and in the United States in 1924 [4]. In South America, it was first reported in Brazil in 1971 and in Argentina in 1983 [5,6]. In the USA, FLS is more commonly found in southern soybean-growing regions where warm temperatures and humid conditions favor disease development [4,7]. However, FLS has recently become more prevalent and severe in northern states with outbreaks reported in Iowa, Ohio, and Wisconsin [8,9,10]. Yield losses in North Central USA were estimated to have been 2.6 million metric tons in the past 10 years (2014 to 2023), which was almost twice the estimated yield loss in the 10 years prior to that (https://loss.cropprotectionnetwork.org/; CPN 2024; accessed on 19 September 2024). The highest yield loss in this region in a single year was recorded in 2018 (1.2 million metric tons) (CPN 2024).

Multiple factors contributed to the rising impact of FLS in North Central USA. The pathogen survives in contaminated soybean seeds and infested soybean residues, with the latter being the main source of primary inoculum in the next season. Warmer winters were reported to increase pathogen survival and contributed to higher disease severity during the following season [9,10,11]. The wider adoption of no-till and conservative-till practice, as well as planting continuous soybeans, increases the amount of soybean debris in the field. The particularly severe disease in 2018 was likely in larger part due to the many rainy days from June to August that year, which was conducive for pathogen sporulation and spore dissemination of this polycyclic disease.

The pathogen primarily infects soybean leaves, causing circular to angular lesions. It can also infect petioles, pods, and seeds. In severe infection, adjacent lesions may coalesce to form larger irregular spots. The lesions may eventually cover more than 30% of the leaf surface and cause premature defoliation [1,2]. Young expanding leaves are more susceptible to infection than mature leaves, but lesions may be visible after the leaflets are fully expanded because the latent period extends up to two weeks. When it is warm and moist, secondary inoculum is produced from lesions on leaves and conidia are spread by air currents and splashing rain, leading to secondary infections. When environmental conditions alternate between dry and wet periods during the production season, a layered pattern of severely diseased leaves and symptom-free leaves can be observed throughout the soybean canopy [12]. Yield loss from FLS is mainly due to reduction in photosynthetic leaf area by necrotic lesions and premature defoliation, resulting in reduced seed weight. Infected seeds have poor germination and seed quality. Yield reductions in the range of 10–60% have been reported [13,14,15,16,17,18].

Multiple strategies have been used to control FLS, including practices that reduce the sources of inoculum (e.g., burying crop residues through tillage, crop rotation, and planting disease-free seeds), planting resistant varieties, and fungicide applications [1,2,7,12,19,20]. The *Rcs3* gene carried on chromosome 16 in soybean cultivar Davis has provided durable resistance against all tested isolates of *C. sojina* since its discovery in the 1980s [21,22], with only occasional small lesions without an enlarged necrotic center caused by some isolates. More recently, two soybean accessions, PI 594891 and PI 594774, were found to confer a high level of resistance to FLS and the resistant loci were mapped to the same region on chromosome 13 [23,24]. Many soybean cultivars grown in North Central USA lack strong host resistance against FLS.

Fungicides with active ingredients belonging to five groups (chloronitriles, methyl benzimidazole carbamate, demethylation inhibitors, succinate dehydrogenase inhibitors, and quinone outside inhibitors (QoIs)) have been labeled for use in soybeans in the USA. QoI fungicides (strobilurins) are broadly used in soybeans due to their broad-spectrum control against many fungal pathogens, including *C. sojina* [20,25]. In the USA, *C. sojina* isolates resistant to QoI fungicides were first identified in Tennessee in 2010 [26]. Since the first report, QoI resistance in *C. sojina* has been reported in over 20 states, including Indiana. QoI-resistant isolates were found to carry the G143A mutation in the mitochondrial cytochrome b (*cytb*) gene [27,28,29,30,31,32,33].

Sexual stage has not been observed in *C. sojina*. In a study that examined mating-type idiomorphs and genetic diversity in six fields from two counties in Arkansas, Kim et al. [34] found that the populations had high genetic diversity based on eight microsatellite loci; both *MAT1-1* and *MAT1-2* idiomorphs existed in *C. sojina*; and their distribution did not significantly deviate from 1:1 ratio at various geographic scales using clone-corrected data. The authors argued that these data suggested a cryptic sexual stage. While significant gametic disequilibrium was detected in these populations, the authors contended that this could be explained by dominant clonal reproduction and dominant genotypes favored by the host and environments. In a study of 62 historical isolates from the USA, China, Brazil, and Nigeria using amplified fragment length polymorphisms, Bradley et al. [35] found that the average genetic similarity between isolates was 0.56 on a scale between 0 and 1, indicating a high level of genetic diversity within this species. On the other hand, Shrestha et al. reported that the *C. sojina* populations from two locations in Tennessee were highly clonal based on 49 single nucleotide polymorphism (SNP) loci, with the majority of the isolates belonging to a single genotype [36].

With FLS rising in North Central USA, where most soybeans are produced in this country, the need for effective FLS control strategies is ever more important. Our knowledge of the population genetics of *C. sojina* is limited. In this study, we investigated the population structure and mating type distribution in *C. sojina* from soybeans in Indiana, USA.

## 2. Materials and Methods

### 2.1. C. sojina Isolates and DNA Extraction

In the summer of 2020, soybean leaves showing FLS symptoms were collected in experimental and commercial fields in 29 counties across Indiana. A map showing the sampled counties is available in [27]. Single-conidial isolates were obtained from 234 leaf lesions and DNA was extracted from all isolates, as previously described [27].

### 2.2. Mating Type Determination

Multiplex PCR reactions using MyTaq™ Red Mix (Meridian Bioscience, Cincinnati, OH, USA) were used to determine the mating-type idiomorph of individual isolates following the protocol of [34]. The primers CsMat1f (TGAGGACATGGCCACCCAAATA) and CsMat1r (AAGAGCCCTGTCAAGTGTCAGT) were used to amplify a portion of the *MAT1-1-1* gene and the primers CsMat2f (TGTTGTAGAGCTCGTTGTTCGCA) and CsMat2r (TCAGACCTTATGAGCTTGAAAGTGCT) were used to amplify a portion of the *MAT1-2* gene. The assay also included the primers Csojina18Sf (TCTCCGTAGGTGAACCTGCG) and Csojina28Sr (TATCCCTACCTGATCCGAGGTCAA) to amplify the internal transcribed region of the ribosomal RNA gene as an internal control. The PCR cycling conditions were an initial denaturation period of 95 °C for 3 min; 39 cycles of denaturation at 95 °C for 30 s; annealing at 64 °C for 30 s; and extension at 72 °C for 30 s; with a final extension at 72 °C for 5 min. The resulting PCR products were separated by electrophoresis in 1.3% agarose gel, stained and visualized under UV light.

### 2.3. Multi-Locus Genotyping Using Single Nucleotide Polymorphism (SNP) Loci

Twenty-seven SNPs loci (Table 1) were used to genotype the *C. sojina* isolates. These loci were also among the 49 nuclear SNPs used in genotyping *C. sojina* isolates from Tennessee in a previous study [36]. In that study, 15 SNPs were able to distinguish all 35 genotypes. Some of the SNPs on the same contigs completely agreed with each other and thus did not provide any additional information. The redundant SNPs were removed in this study. The 27 SNPs used in this study are located on 8 out of 12 contigs of GenBank genome assembly ASM429982v1 (Table S1). Genomic DNA was sent to Floodlight Genomics (Knoxville, TN, USA) for targeted sequencing. Primers were designed using the 100 bp flanking sequences on both sides and multiplex PCR was used to amplify the target sequences. Adapters with barcode sequences were ligated to amplified PCR products and sequenced on an Illumina HiSeq3000 device (San Diego, CA, USA). After demultiplexing, sequences were mapped to the reference contigs (GenBank Accession ASM429982v1). Genotypes were assigned for loci with at least 20× coverage.

### 2.4. Data Analysis

Isolates were assigned to multi-locus genotypes (MLGs) based on the 27 SNP markers. All further analyses were performed using the clone-corrected data set. Analyses regarding alleles frequence and diversity index were performed using POPGENE [37]. The population structure was assessed by Bayesian clustering in Structure version 2.3.4 [38]. The parameters were an admixture model, 1,000,000 burn-in, followed by 1,000,000 Markov Chain Monte Carlo simulations with 30 replications for each K (ranging from K = 1 to K = 49). The online tool Structure Selector (https://lmme.ac.cn/StructureSelector/ (accessed on 11 April 2024)) was used to select the optimal K from the results obtained from the Structure [39]. A genetic distance was calculated for all MLGs and used for the principal coordinate analysis (PCoA) in GENALEX version 6.502 [40]. The unweighted pair group method with arithmetic mean (UPGMA) analysis was performed using Euclidian distance and 10,000 bootstrap in Past software version 4.03 [41]. The genotype accumulation curve, the standardized index of association ($\bar{r}$_d_), and Nei’s gene diversity were accessed using the poppr package in R software version 4.1.2 [42,43]. Linkage disequilibrium between all pairs of loci and the analysis of molecular variance (AMOVA) between populations were performed in Arlequin version 3.5.2.2 [44]. The minimum spanning tree was constructed using PopART [45]. A Chi-square (*X*^2^) goodness-of-fit test was employed to determine if the distribution of the mating-type idiomorphs deviated significantly from the expected 1:1 ratio using GraphPad Prism 8 software.

## 3. Results

### 3.1. Mating Type

A total of 594 single-conidial isolates were obtained from 234 FLS lesions with 1 to 5 isolates per lesion. The mating type was determined for all isolates. The genotype and QoI resistance were determined for multiple isolates from a subset of lesions (data not shown and [27,46]). Isolates from the same lesions always had the same genotype, mating-type idiomorph, and QoI resistance/susceptibility profile, indicating that each lesion was caused by a single infection. In the following sections, the results are presented on one representative isolate per lesion.

Only one mating-type idiomorph was found in each isolate. Isolates from 186 lesions (79.5%) had the *MAT1-1* idiomorph and isolates from 48 (20.5%) lesions had the *MAT1-2* idiomorph. Isolates with the *MAT1-1* idiomorph were found in all 29 counties and isolates with the *MAT1-2* idiomorph were found in 16 out of 29 counties (Table 1).

### 3.2. Multi-Locus Genotypes

Based on the 27 SNP markers, the 234 isolates belonged to 49 MLGs (Table 2). Twenty-six of the 27 SNPs showed polymorphisms in the Indiana population with minor allele frequency ranging from 0% to 0.449%. Nineteen SNPs had an effective allele number more than 1.5 (Table S2). The genotype accumulation curve showed that 11 SNPs were able to identify more than 40 MLGs, and 20 SNPs were able to detect all the 49 MLGs (Figure S1).

**Figure 1:**
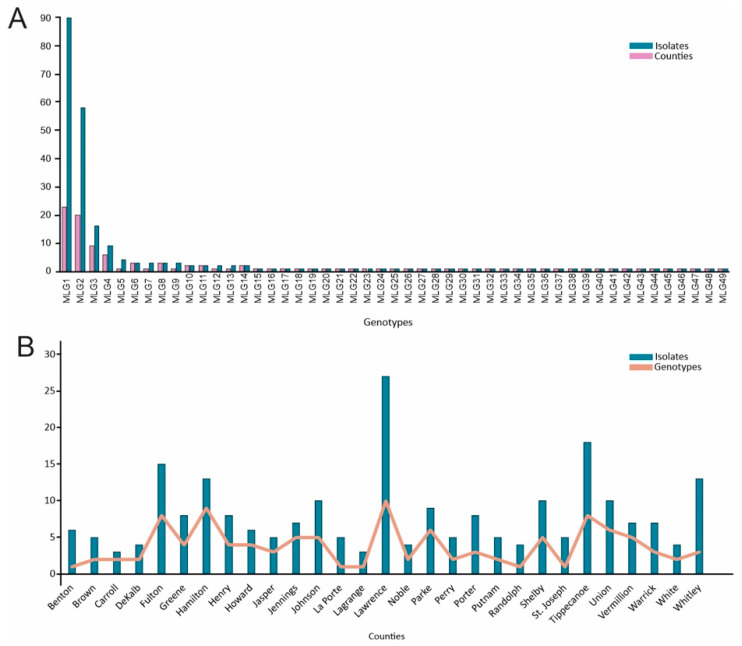
Distribution of *Cercospora sojina* genotypes in Indiana counties. (**A**) Number of counties in which each multi-locus genotype (MLG) was found and number of isolates in each MLG. (**B**) Number of MLGs found and isolates collected in each county.

MLG1 and MLG2 dominated the Indiana population with 90 isolates (38.5%) and 58 isolates (24.8%), respectively. Two to 16 isolates were found in 12 MLGs and 35 MLGs had only one isolate each (Table 2). MLG1 was found in 23 counties and MLG2 was found in 20 counties. The number of MLGs found in individual counties ranged from 1 to 10, and two or more MLGs were found in 24 counties (Figure 1).

Only one mating-type idiomorph was found in each MLG. Twenty-two MLGs had the *MAT1-1* idiomorph and 27 MLGs had the *MAT1-2* idiomorph. Both MLG1 and MLG2, the two MLGs with the most isolates, had the *MAT1-1* idiomorph. The most common MLG with the *MAT1-2* idiomorph was MLG4 with nine isolates (Table 2). Using clone-corrected data, the null hypothesis of equal distribution of mating-type idiomorphs was not rejected at *p* = 0.05 in 22 out of 24 counties in which multiple MLGs were found, and in Indiana as a whole. The null hypothesis was rejected only in Greene County and Jennings County. Four and five MLGs were found in Greene County and Jennings County, respectively, and they all had the *MAT1-1* idiomorph (Table 1).

The QoI resistance of these isolates was determined previously [27]. Thirty MLGs contained QoI-resistant isolates and 22 MLGs contained QoI-sensitive isolates, with three MLGs containing both types of isolates. Of the 22 MLGs with the *MAT1-1* idiomorph, 11 MLGs contained only QoI-resistant isolates and eight MLGs contained only QoI-sensitive isolates, with the remaining three MLGs (MLG1, MLG3 and MLG8) containing both types of isolates. Of the two MLGs with the most isolates, MLG1 was dominated by QoI-resistant isolates (88 out of 90 isolates), and MLG2 contained only QoI-sensitive isolates. Of the 27 MLGs with the *MAT1-2* idiomorph, 16 MLGs contained only QoI-resistant isolates and 11 MLGs contained only QoI-sensitive isolates. The most common MLG with the *MAT1-2* idiomorph, MLG4, contained only QoI-sensitive isolates (Table 2).

### 3.3. Population Structure

**Figure 2:**
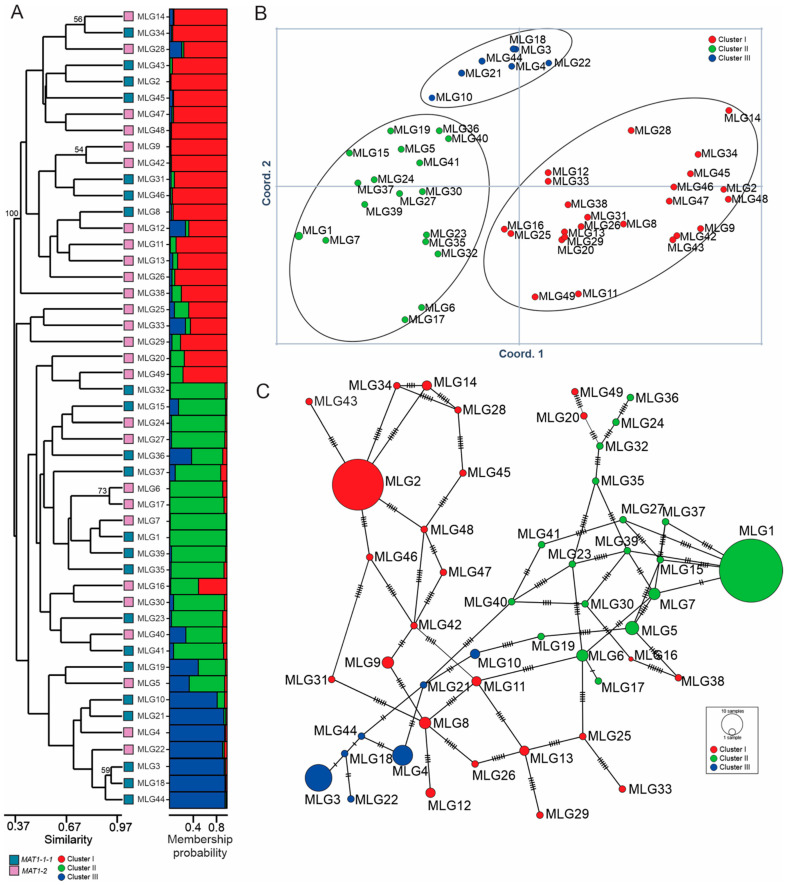
Population structure of *Cercospora sojina* population from Indiana, USA. (**A**) Right side, Bayesian cluster analysis assigned the 49 multi-locus genotypes (MLGs) into 3 clusters. The relative abundance of red, green, and blue color on the horizontal bars indicates the likelihood of individual MLGs belonging to a particular cluster. Left side, UPGMA dendrogram. Numbers indicate bootstrap support in percentile. Bootstrap support values above 50% were labeled above the respective branches. (**B**) Principal coordinate analysis (PCoA). (**C**) Minimum spanning tree. The areas of the circles are proportional to the number of isolates in each MLG. The small perpendicular line segments on the connecting lines represent additional differences between neighboring MLGs.

The 49 MLGs most likely belonged to three clusters based on the Bayesian analysis (Figure 2A, right side, and Figure S2). PCoA supported the result from the Bayesian analysis (Figure 2B). Cluster I contained 24 MLGs, cluster II contained 18 MLGs, and 7 MLGs belonged to cluster III. Both the *MAT1-1* and *MAT1-2* idiomorphs were found in each cluster. In the UPGMA dendrogram (Figure 2A, left side), 18 of the 24 MLGs in cluster I were grouped together with 100% bootstrap support. The bootstrap support values for other branches in the dendrogram were low (<50%) except for four terminal branches. While most MLGs in cluster II grouped together in the UPGMA dendrogram, this grouping had low bootstrap support (<50%). A similar result was observed for cluster III. In the minimum spanning tree (Figure 2C), most MLGs in clusters I, II, and III grouped together but four MLGs in cluster I, MLG 16, MLG20, MLG38, and MLG49 grouped with cluster II instead.

**Figure 3:**
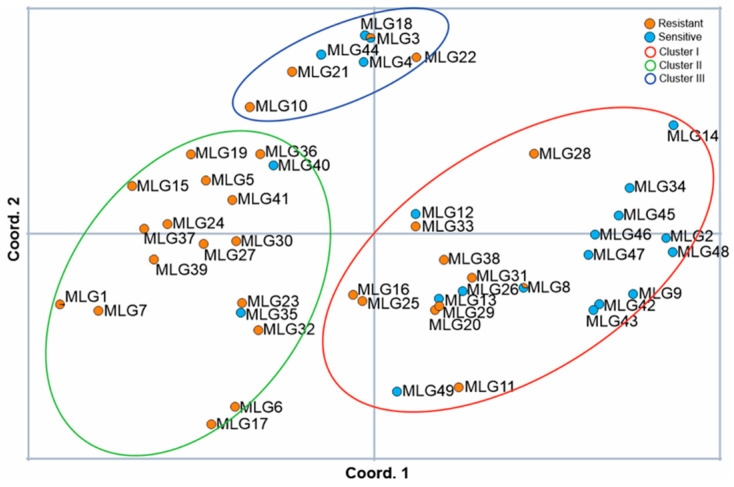
Principal coordinate analysis (PCoA) showing the distribution of QoI-resistant and QoI-sensitive multi-locus genotypes (MLGs) in individual clusters.

Cluster II was dominated by QoI-resistant MLGs. Of the 18 MLGs in cluster II, only MLG35 and MLG40 contained only QoI-sensitive isolates. Of the other 16 MLGs, 15 only contained QoI-resistant isolates and MLG1 was dominated by QoI-resistant isolates (Figure 3).

Nei’s gene diversity [43] of the Indiana population was 0.393. MLGs with the *MAT1-1* idiomorph had similar genetic diversity as MLGs with the *MAT1-2* idiomorph, and the QoI-resistant MLGs had slightly less genetic diversity than the QoI-sensitive MLGs (Table 3). In the 24 counties where two or more MLGs were found, Nei’s gene diversity ranged from 0.247 in Porter County to 0.593 in White County. Based on AMOVA, geographic location (counties) did not have a significant effect on the *C. sojina* genetic composition (*F_ST_* = −0.083, *p*-value = 0.998).

### 3.4. Linkage Disequilibrium

Test for multi-locus gametic disequilibrium by $\bar{r}$_d_ showed that the Indiana population as a whole was in significant disequilibrium ($\bar{r}$_d_ = 0.029, *p*-value = 0.001). In the 17 counties where three or more MLGs were found, 11 were in significant disequilibrium at *p*-value of 0.05 (Table 3). The 27 SNPs are located on eight contigs based on GenBank genome assembly ASM429982v1 of a *C. sojina* isolate [47]. Excluding SNP25, which did not show diversity in the Indiana population, linkage disequilibrium was tested between all locus pairs. Of the 42 locus pairs on the same contigs, 14 pairs (33.3%) were in significant disequilibrium at *p*-value of 0.05. Of the 283 locus pairs on different contigs, 50 (17.7%) were in significant disequilibrium (Table 4). 

## 4. Discussion

In this study, we examined the population structure and mating type distribution in the *C. sojina* population from Indiana, USA. Based on 27 SNP markers, 234 isolates were grouped into 49 MLGs. Only one mating-type idiomorph was found in each isolate and each MLG. Twenty-two MLGs had the *MAT1-1* idiomorph and 27 MLGs had the *MAT1-2* idiomorph. The two most common MLGs, MLG1 and MLG2, had 90 and 58 isolates, respectively. MLG1 was dominated by QoI-resistant isolates and MLG2 contained only QoI-susceptible isolates.

Shrestha et al. [36] used 49 nuclear SNP markers and the mitochondrial QoI resistance locus to genotype 186 isolates, including 161 isolates from Tennessee collected in 2015, 10 isolates from Tennessee collected in 2007, and 15 isolates from other states collected between 2006 and 2009. The 186 isolates were grouped into 35 genotypes, from G01 to G35. Because the 27 SNPs used in our study were included in the 49 SNPs used in their study (Table S1), we were able to compare our results with their findings. Since we used a smaller number of SNPs, one MLG in our study could match multiple genotypes in their results. MLG1 matched G22, G23, and G24, MLG3 matched G32, and MLG8 matched G11 (Table 2). No other MLGs matched any genotype in their study. G22 was the most dominant genotype in the Tennessee population collected in 2015 (130 out of 161 isolates). Together, MLG1 (G22/G23/G24) contained 141 out of 161 Tennessee isolates collected in 2015 and 1 out of 10 Tennessee isolates collected in 2007. Of the 142 Tennessee isolates belonging to MLG1, 131 were QoI-resistant and 11 were QoI-susceptible, similar to the findings in Indiana showing that MLG1 was dominated by QoI-resistant isolates.

Only 1 in 10 Tennessee isolates collected in 2007 belonged to MLG1, and none of the 15 isolates from other states collected between 2006 to 2009 belonged to MLG1 [36], suggesting that the dominance of MLG1 has increased since 2007. Given that Tennessee and Indiana are not neighboring states and the isolates from Indiana and Tennessee were collected at least five years apart, MLG1 had likely been a dominant genotype in *C. sojina* populations in a wide region for many years. This contention was corroborated by our preliminary findings that showed that MLG1 was also the most dominant genotype in the *C. sojina* population collected in Kentucky between 2017 and 2022 (unpublished). Kentucky is located between Tennessee and Indiana.

MLG1 was observed in Tennessee as a single isolate belonging to G24 in 2007. By 2015, 10 out of 161 isolates from Tennessee belonged to G24, one isolate belonged to G23, and 131 isolates belonged to G22. All the isolates belonging to G24 were QoI-sensitive, and the isolates belonging to G23 and G24 were all QoI-resistant. G23 differed from G24 only by the QoI-resistance locus, while G22 differed from G23 by another locus, L15 [36]. Based on these analyses, it is likely that isolates belonging to G24 gained the G143A mutation in the mitochondrial *cytb* gene, either through mutation or hyphal anastomosis with a QoI-resistant isolate, and became G23. A mutation at the L15 locus led to isolates belonging to G22. Isolates with the G22 genotype likely had a competitive advantage over isolates with the G23 genotype (e.g., better sporulation, favored by the environments or the host, etc.), and became much more common. Of the 90 Indiana isolates belonging to MLG1, 88 were QoI-resistant and two were QoI-sensitive. We hypothesize that if these isolates were genotyped with the 22 additional SNPs that were used by Shrestha et al. [36] but not in our study (or only the L15 locus), most or all the QoI-resistant isolates would have the G22 genotype and the two QoI-sensitive isolates would have the G24 genotype.

MLG2, the genotype with the second most isolates in Indiana, did not match any genotype from Tennessee collected between 2007 and 2015, or the small number of isolates from other states collected between 2006 and 2009. This suggests that MLG2 either was local in Indiana, appeared more recently, or has been suppressed in Tennessee, likely due to the extensive use of QoI fungicides in soybean production there as MLG2 contained only QoI-sensitive isolates in Indiana. However, more data on *C. sojina* populations and additional analysis are needed to address this issue. Our preliminary results showed that MLG2 was also the second most common genotype found in isolates collected in Kentucky between 2017 and 2022 and exclusively contained QoI-sensitive isolates (unpublished).

In our study, only one mating-type idiomorph was found in each isolate and each MLG. Shrestha et al. [34] reported both mating-type idiomorphs in 15 out of 35 genotypes, including 10 genotypes each containing a single isolate, suggesting that both mating-type idiomorphs were found in the same isolate in at least 10 cases. This contradicted our findings and the findings by Kim et al. [34]. When examining the mating type distribution and genetic diversity in 132 *C. sojina* isolates from Arkansas, Kim et al. [34] found only one mating-type idiomorph in each isolate. Heterokaryon could be a plausible explanation for the detection of both mating-type idiomorphs in the same isolate on rare occasions.

Like most *Cercospora* spp., sexual stage has not been observed in *C. sojina*. Phylogenetically, *Cercospora* spp. form a strongly-supported monophyletic group within the teleomorphic genus *Mycosphaerella* [48]. Indeed, the few *Cercospora* spp. in which teleomorphs were found belonged to *Mycosphaerella* [49,50]. Whether *C. sojina* has a cryptic sexual stage has important implications for disease management because sexual recombination allows the pathogen to produce genetically diverse offsprings to overcome host resistance and with better fitness for the ever-changing environments [51].

In the absence of an observed sexual stage, random mating can be inferred from the equal distribution of mating-type idiomorphs [52]. The rationale is that, in a strictly asexually reproducing fungal species, genetic drift would lead to the dominance of one mating-type idiomorph over the other, and in extreme cases, the disappearance of one mating-type idiomorph all together. For example, in *Fusarium virguliforme*, the fungal pathogen that causes sudden death syndrome in soybeans, only the *MAT1-1* idiomorph was found [53]. In Arkansas, Kim et al. [34] found that the distribution of mating-type idiomorphs did not significantly deviate from 1:1 ratio in *C. sojina* collected in six individual soybean fields using clone-corrected data. In our study, 22 MLGs in the Indiana population had the *MAT1-1* idiomorph and 27 MLGs had the *MAT1-2* idiomorph, which did not significantly deviate from 1:1 ratio. At the county level, the distribution of mating-type idiomorphs did not significantly deviate from 1:1 ratio in 22 out of 24 counties where multiple MLGs were found.

Another indirect evidence of sexual reproduction is the lack of linkage among genetic loci [52]. The index of association ($\bar{r}$_d_) tests the linkage among multi-loci, and the Indiana population as a whole was in significant linkage disequilibrium based on $\bar{r}$_d_, suggesting clonal reproduction. Given our knowledge of FLS disease cycle, even if a cryptic sexual stage exists, sexual reproduction is likely to be much less frequent than clonal reproduction. At the county level, the null hypothesis of equilibrium among loci was not rejected in 6 out of 17 counties where three or more MLGs were found. It is notable that in Warrick County and Whitley County, the $\bar{r}$_d_ values were negative. Linkage can also be tested between locus pairs. The 27 SNPs are located on eight contigs based on GenBank genome assembly ASM429982v1 [47]. For loci on the same contigs, only 33.3% locus pairs were in significant disequilibrium. A much lower level of linkage disequilibrium was observed in locus pairs on different contigs. The lack of linkage disequilibrium for most locus pairs, even if they are located on the same contigs, is the strongest evidence suggesting the existence of a cryptic sexual stage. These findings point to the potential existence of a sexual stage. On the other hand, the evidence for the existence of a cryptic sexual stage described above did not rule out the possibility that *C. sojina* lost its sexual reproduction recently and evolution has not had enough time to cause prevalent linkage disequilibrium and unequal distribution of mating-type idiomorphs.

This report is our first step toward characterizing the global population structure of *C. sojina*. The population structure of a pathogen reflects the evolution potential of the pathogen [54]. It can also aid breeding efforts by selecting genetically diverse, representative strains used for screening soybean germplasm and breeding lines.

## Figures and Tables

**Table 1:** Mating type distribution in the *Cercospora sojina* population from Indiana, USA.

County	# of Isolates	Mating Type				χ2 ^a^	*p*-Value ^b^
		Uncorrected		Clone-Corrected			
		*MAT1-1*	*MAT1-2*	*MAT1-1*	*MAT1-2*		
Benton	6	6	0	1	0	-	-
Brown	5	5	0	2	0	2.000	0.157
Carroll	3	2	1	1	1	0.000	1.000
DeKalb	4	2	2	1	1	0.000	1.000
Fulton	15	8	7	2	6	2.000	0.157
Greene	8	8	0	4	0	4.000	0.046 *
Hamilton	13	11	2	7	2	2.778	0.096
Henry	8	7	1	3	1	1.000	0.317
Howard	6	5	1	3	1	1.000	0.317
Jasper	5	5	0	3	0	3.000	0.083
Jennings	7	7	0	5	0	5.000	0.025 *
Johnson	10	9	1	4	1	1.800	0.180
La Porte	5	5	0	1	0	-	-
Lagrange	3	3	0	1	0	-	-
Lawrence	27	10	17	2	8	3.600	0.058
Noble	4	4	0	2	0	2.000	0.157
Parke	9	8	1	5	1	2.667	0.102
Perry	5	4	1	1	1	0.000	1.000
Porter	8	6	2	1	2	0.333	0.564
Putnam	5	5	0	2	0	2.000	0.157
Randolph	4	4	0	1	0	-	-
Shelby	10	8	2	3	2	0.200	0.655
St. Joseph	5	5	0	1	0	-	-
Tippecanoe	18	15	3	5	3	0.500	0.480
Union	10	7	3	4	2	0.667	0.414
Vermillion	7	5	2	3	2	0.200	0.655
Warrick	7	5	2	1	2	0.333	0.564
White	4	4	0	2	0	2.000	0.157
Whitley	13	13	0	3	0	3.000	0.083
Total	234	186	48	22	27	0.510	0.475

^a^ χ2 analysis was conducted using clone-corrected data. ^b^ *p*-values less than 0.05 are labeled with an asterisk.

**Table 2:** Multi-locus genotypes found in the *Cercospora sojina* population from Indiana, USA.

ID	Multi-Locus Genotype	# of Isolates	QoI Resistance ^a^		Mating Type		Corresponding Genotypes in [36]
			R	S	*MAT1-1*	*MAT1-2*	
MLG1	TAGAATCGAGCGGGCATGAGACCGTTC	90	88	2	90	0	G22/G23/G24
MLG2	TGGGATTAAATGAATGAAAGATAATCC	58	0	58	58	0	-
MLG3	TGTGCTCGGGCGAACGAAAAACCGTCC	16	9	7	16	0	G32
MLG4	CGTACCCGGACGAACGAAAGACCGTCC	9	0	9	0	9	-
MLG5	CAGAACCGGGCGGACGAAAGACCGTTC	4	4	0	0	4	-
MLG6	CAGAATCGAGCCGGCGTGAGATAGTTC	3	3	0	0	3	-
MLG7	CAGAATCGAGCGGGCGTGAGACCGTTC	3	3	0	0	3	-
MLG8	CGTAACTGGGCCAGTGAGGGATAGTCC	3	1	2	3	0	G11
MLG9	CGGAACTAGATCAATGAGGGATAGTCC	3	0	3	0	3	-
MLG10	TGTACCCGGACGAGCGAGAGACCGTTC	2	2	0	2	0	-
MLG11	CGGAATTGAGCCGGTGAGAGATAGTCC	2	2	0	0	2	-
MLG12	CGTAACTGGGCCAGTGAGAAACCGTCC	2	0	2	0	2	-
MLG13	CATACTCGAGCCAGTGAGAGATAGTCC	2	0	2	0	2	-
MLG14	TGTGCCTAGGTGAATGAAAGGTAATCC	2	0	2	0	2	-
MLG15	TATAATCGGGCGGACAAGAGACCGTTC	1	1	0	1	0	-
MLG16	CAGAACTAAGTGGATGTGAGACCGTCC	1	1	0	0	1	-
MLG17	CAGAATCGAGCCGGCATGAGATAGTTC	1	1	0	0	1	-
MLG18	TGTGCTCGGGCGAACGAAAGACCGTCC	1	0	1	1	0	-
MLG19	TAGACCCGGGCGAGCGTAAGACCGTTC	1	1	0	1	0	-
MLG20	TGGACTTAAGCGAATATGAGATAGTTC	1	1	0	0	1	-
MLG21	TGTACCCGGGCGAACGAGAGACCGTCC	1	1	0	1	0	-
MLG22	CGTGCTCAGGCGAACGAAAGACCGTCC	1	1	0	0	1	-
MLG23	TAGAACCGGGCGGGCGTGGGATAGTCC	1	1	0	1	0	-
MLG24	TGTAATCAAGCGGACATGAGGCCGTTC	1	1	0	0	1	-
MLG25	CAGACTCGAGCCAACGAGAAATAGTTC	1	1	0	0	1	-
MLG26	CGTACCCGAGCCAGTAAGGAATAGTCT	1	0	1	0	1	-
MLG27	TAGAATCAAGCGGACAAGGGACCGTCC	1	1	0	0	1	-
MLG28	TGTGCCCAAGCGAATGAAAGATAGTTC	1	1	0	0	1	-
MLG29	CAGACCCGAGCCAATATAAGATAGTCC	1	1	0	0	1	-
MLG30	CGGAACCAAGCGGACGTGAGACCGTCC	1	1	0	0	1	-
MLG31	TGTAATTGGGTGGGTGAGGGATAATCC	1	1	0	1	0	-
MLG32	TGGAATCAAGCGGACATGAGATAGTTC	1	1	0	1	0	-
MLG33	CGGACCCGAGCGAGCGAAAAATAGTCC	1	1	0	0	1	-
MLG34	TATGCTTAAGTGAATGAAAGGTAGTCC	1	0	1	1	0	-
MLG35	TGTAATCGAGCGGGCATGAGATAGTCC	1	0	1	1	0	-
MLG36	TGTAACCAAACGGACAAAAGACCGTTC	1	1	0	1	0	-
MLG37	TATAATCGAGCGGGCGAGAAACCATTC	1	1	0	1	0	-
MLG38	CAGGACTAGACGGATGAGAGACAGTTC	1	1	0	0	1	-
MLG39	TGGAATCGAGCGGGCGTGAGACCGTCC	1	1	0	1	0	-
MLG40	TGGAACCAGGCGAACGTGGGACCGTCC	1	0	1	0	1	-
MLG41	TGGAATCAGGCGGACGTGGGGCCGTCC	1	1	0	1	0	-
MLG42	CGGAATTAGGTCAATGAGAGATAGTCC	1	0	1	0	1	-
MLG43	TGGGATTAAACCGATGTAAGGTAATCC	1	0	1	1	0	-
MLG44	TGTACTCGGGCGAACGAAAGACCGTCC	1	0	1	1	0	-
MLG45	TGGGCTCAAACCAATGAAAAATAGTCC	1	0	1	1	0	-
MLG46	TGGGATTAGGTGGATGAAGGATAGTCC	1	0	1	1	0	-
MLG47	CGGGACTAAGCCAACGAAAGATAGTCC	1	0	1	0	1	-
MLG48	CGTGATTAAACCAATGAAAGATAGTCC	1	0	1	0	1	-
MLG49	CGGAATTGAACGAGTATGAAATAGTTC	1	0	1	0	1	-
Total		234	134	100	186	48	

^a^ Based on results from [27].

**Table 3:** Genetic diversity within populations of *Cercospora sojina* from Indiana, USA.

Population	N ^a^	G ^b^	H ^c^	$\bar{r}$_d_ ^d^	$p.\bar{r}$_d_ ^e^
Benton	6	1	-	-	-
Brown	5	2	0.556	-	-
Carroll	3	2	0.333	-	-
DeKalb	4	2	0.481	-	-
Fulton	15	8	0.435	0.025	0.033
Greene	8	4	0.432	0.074	0.041
Hamilton	13	9	0.409	0.047	0.002
Henry	8	4	0.444	0.063	0.047
Howard	6	4	0.438	0.051	0.084
Jasper	5	3	0.321	0.096	0.167
Jennings	7	5	0.370	0.127	0.003
Johnson	10	5	0.422	0.120	0.001
La Porte	5	1	-	-	-
Lagrange	3	1	-	-	-
Lawrence	27	10	0.397	0.081	0.001
Noble	4	2	0.481	-	-
Parke	9	6	0.430	0.031	0.050
Perry	5	2	0.481	-	-
Porter	8	3	0.247	0.167	0.094
Putnam	5	2	0.444	-	-
Randolph	4	1	-	-	-
Shelby	10	5	0.452	0.098	0.001
St. Joseph	5	1	-	-	-
Tippecanoe	18	8	0.410	0.035	0.010
Union	10	6	0.430	0.036	0.040
Vermillion	7	5	0.363	0.059	0.034
Warrick	7	3	0.420	−0.037	0.747
White	4	2	0.593	-	-
Whitley	13	3	0.494	−0.011	0.484
All	234	49	0.393	0.029	0.001
*MAT1-1*	186	22	0.380	-	-
*MAT1-2*	48	27	0.385	-	-
QoI-resistant	134	30	0.360	-	-
QoI-sensitive	100	22	0.393	-	-

^a^ Number of isolates; ^b^ Number of multi-locus genotypes (MLGs); ^c^ Nei’s gene diversity [43]; ^d^ Standardized Index of Association; ^e^ *p*-value for $\bar{r}$_d_.

**Table 4:** Linkage disequilibrium between pairs of SNP locus in the 49 MLGs of *Cercospora sojina* population from Indiana, USA.

Contig ^a^	SNP ^b^																										
I	SNP11																										
I	SNP24	+ ^c^																									
I	SNP21	+	+																								
II	SNP17	−	−	−																							
III	SNP26	+	−	−	−																						
III	SNP19	−	−	−	−	+																					
III	SNP27	−	−	−	−	−	−																				
VI	SNP3	−	−	−	+	−	−	−																			
VI	SNP13	−	−	−	+	+	−	−	−																		
VI	SNP5	−	−	−	+	−	−	+	+	+																	
VI	SNP7	+	+	−	−	−	−	−	−	−	−																
VI	SNP15	+	−	−	−	−	−	−	−	−	−	+															
VII	SNP9	−	−	−	+	−	+	−	+	−	−	−	−														
VII	SNP16	−	−	−	+	+	−	+	−	−	−	−	−	+													
VII	SNP6	−	−	−	−	−	−	+	−	−	−	−	−	−	−												
VII	SNP23	−	−	−	−	−	−	−	−	−	−	+	+	+	−	−											
VII	SNP22	−	−	−	−	−	−	−	−	−	−	+	+	+	−	−	+										
VIII	SNP12	−	−	−	−	−	−	−	−	−	−	−	+	−	−	−	+	+									
VIII	SNP1	−	−	−	−	−	−	−	−	−	−	−	−	−	−	−	−	−	+								
VIII	SNP14	−	−	−	−	−	−	+	−	−	−	−	−	−	−	−	−	−	−	−							
VIII	SNP8	+	−	+	−	−	−	−	−	−	−	+	+	−	−	−	−	−	−	−	+						
VIII	SNP2	−	−	−	−	+	−	−	−	+	−	−	−	−	−	−	−	−	−	−	−	−					
VIII	SNP10	−	−	−	−	−	−	−	−	−	−	+	+	−	−	−	−	−	−	−	−	−	−				
X	SNP20	−	−	−	−	−	−	+	−	−	+	−	−	−	−	−	−	−	−	−	+	−	−	−			
XII	SNP18	−	−	−	+	−	−	−	−	+	+	−	−	−	+	−	−	−	−	−	+	−	−	−	−		
XII	SNP4	−	+	+	+	+	−	−	−	−	−	+	+	−	+	−	−	−	−	−	+	+	−	+	−	+	
	SNP ^b^	SNP11	SNP24	SNP21	SNP17	SNP26	SNP19	SNP27	SNP3	SNP13	SNP5	SNP7	SNP15	SNP9	SNP16	SNP6	SNP23	SNP22	SNP12	SNP1	SNP14	SNP8	SNP2	SNP10	SNP20	SNP18	SNP4
	Contig ^a^	I	I	I	II	III	III	III	VI	VI	VI	VI	VI	VII	VII	VII	VII	VII	VIII	VIII	VIII	VIII	VIII	VIII	X	XII	XII

^a^ Based on GenBank genome assembly ASM429982v1. ^b^ SNPs were arranged by contigs. For multiple SNPs on the same contig, they were arranged from left to right on contig assembly. ^c^ Linkage disequilibrium tests between loci on the same contigs are in light gray background. +, locus pairs are in significant linkage disequilibrium at *p*-value 0.05; −, locus pairs are not in significant linkage disequilibrium at *p*-value 0.05.

## Data Availability

The data presented in this study are available in the paper and Supplemental Materials.

## Supplementary Materials

The following supporting information can be downloaded at: https://www.mdpi.com/article/10.3390/jof10110802/s1, Figure S1: Number of multi-locus genotypes (MLGs) detected in *Cercospora sojina* population from Indiana, USA, by randomly sampling of a subset of 26 polymorphic SNPs markers; Figure S2: Delta K graph for each K cluster; Table S1: Summary of the 27 SNP loci used in this study; Table S2: Descriptive statistics of the 27 SNPs based on 49 multi-locus genotypes in the *Cercospora sojina* population from Indiana, USA.
