# Supplementary material for: Population Structure and Mating Type Distribution of Cercospora sojina from Soybeans in Indiana, United States
Source: J Fungi (Basel). 2024 Nov 19;10(11):802. doi: 10.3390/jof10110802 (PMC11595534; doi:10.3390/jof10110802)
Supplement: Supplementary file 1 [file jof-10-00802-s001.zip › jof-3315887-supplementary.pdf]

Supplemental tables

Table S1. Summary of the 27 SNP loci used in this study.

Locus ID	Genome location[a]	Alleles	Forward Primer (5'-3')	Reverse Primer (5'-3')	Corresponding locus ID in [36]
SNP1	Contig8:1209315	C/T	GTGGAAATATGCGACTGCAA	TTGCTCACAAGATCGAACCA	L01
SNP2	Contig8:4281863	A/G	TTCGTCTCTCCGGTCTTGTT	CATCCCTCGGAATTCCACT	L02
SNP3	Contig6:441795	G/T	GAGCCGATCTGGTCTTTGAG	CCCGTCATCCTACGAAAATG	L04
SNP4	Contig12:3601218	A/G	AAGCCTGCTCGCAACTTTTA	ATCACTCCGAAAGCCACAAC	L05
SNP5	Contig6:2780356	A/C	AGATCCAGGATGACCAGCAG	CATGACCATGGTGCACAGA	L06
SNP6	Contig7:1906877	C/T	CCATAACGCAGGGTACACG	ACGAGATACCCACGATCGAC	L07
SNP7	Contig6:2997421	C/T	AATGCCACACAGGGTCTGAT	GACTGACTTTGGCGTTGTCC	L08
SNP8	Contig8:2670243	A/G	GCTTACGGAACAGACCAGGA	CGATTGCAGCACTAGGTGTC	L09
SNP9	Contig7:601835	A/G	TTGACGGTGTCGAACTTCAG	GGCACAAGCTGCACCAC	L10
SNP10	Contig8:5055700	A/G	GAGGGAATGGGGATGGATTA	GAGCGTTTCACTGCCCATAG	L11
SNP11	Contig1:1145373	C/T	CTGTTGAGGTTGGAGGCTGT	CAGCACTTGCAGTCGAACTC	L12
SNP12	Contig8:722450	C/G	CAAGCAATCCGCTCTCAGTC	CACCTGCTGCTGCTCCTAT	L16
SNP13	Contig6:2111999	A/G	TGTGAAGCTTGAGCCCTTTT	CCGTATAGTCCGACCTGGAA	L17
SNP14	Contig8:2288225	A/G	TACCGAGTTGAGCGTCAATG	GGTCGGACGTCTTTGAAGG	L25
SNP15	Contig6:3335864	C/T	TCCCAGATCGAGACAAGGTT	TCGTCTCCCACACCTCTGA	L26
SNP16	Contig7:1079460	A/G	TGCCCTTCAGTACCAGTGTG	ATCTCGATGGCACGACAGAT	L27
SNP17	Contig2:4508057	A/T	CCTCATCGTGATGGTCACCT	GGCGGTGTACTCGAGCTATT	L28
SNP18	Contig12:3477121	A/G	AGTGAAGGAGTCGCGTCCAG	GCAGAGGTGCAAGAGAGGTG	L32
SNP19	Contig3:3774971	A/G	TCGATGAGGATGTTGTCGAG	CAGCAGCAGCAGAAGAAGG	L34
SNP20	Contig10:1537862	A/G	GATGTTCCGTGGACTCATGTC	GACACCAGCACCAATCAAGA	L35
SNP21	Contig1:1972181	A/G	CCTGATCACGACGCACAA	GCCTTTGAATGGGTTCATGT	L36
SNP22	Contig7:2636841	C/T	TCCTGTGATCATCGGTCGTA	TGTAAGCCCAGAGGGAAGTG	L37
SNP23	Contig7:2614761	A/C	GAATTGGAGCCCCATGAAG	GTTTTCGCTCCTGTGTAGGC	L38
SNP24	Contig1:1554264	A/G	CTGGCTAACTATCTCCCAGCA	GACTTCGCAAAGGACTGGAC	L40
SNP25	Contig4:2025085	C/T	CGACTCCATAGCCAATGACC	GGGTGCTGGTGTACAAATC	L42
SNP26	Contig3:3202129	C/T	AAGGTCACCACTGCTCTTGG	GCTATGACCTTTCCCCTCGT	L44
SNP27	Contig3:4399650	C/T	ATGCGGTAGGACCATTCAAG	CGTGAAGCTAGCCAGTCGAT	L46

[a] Based on GenBank genome assembly ASM429982v1.

Table S2. Descriptive statistics of the 27 SNPs based on 49 multi-locus genotypes in the *Cercospora sojina* population from Indiana, USA.

Locus ID	Major allele	Minor allele	Minor allele frequency	Na[a]	Ne[b]	h[c]	I[d]
SNP1	T	C	0.449	2	1.979	0.495	0.688
SNP2	G	A	0.327	2	1.785	0.440	0.632
SNP3	G	T	0.429	2	1.960	0.490	0.683
SNP4	A	G	0.265	2	1.639	0.390	0.579
SNP5	A	C	0.367	2	1.869	0.465	0.658
SNP6	T	C	0.408	2	1.935	0.483	0.676
SNP7	C	T	0.347	2	1.829	0.453	0.646
SNP8	G	A	0.449	2	1.979	0.495	0.688
SNP9	A	G	0.429	2	1.960	0.490	0.683
SNP10	G	A	0.204	2	1.481	0.325	0.506
SNP11	C	T	0.163	2	1.376	0.273	0.445
SNP12	G	C	0.306	2	1.739	0.425	0.616
SNP13	A	G	0.449	2	1.979	0.495	0.688
SNP14	A	G	0.367	2	1.869	0.465	0.658
SNP15	C	T	0.429	2	1.960	0.490	0.683
SNP16	G	A	0.245	2	1.587	0.370	0.557
SNP17	A	T	0.367	2	1.869	0.465	0.658
SNP18	G	A	0.388	2	1.904	0.475	0.668
SNP19	A	G	0.184	2	1.428	0.300	0.477
SNP20	G	A	0.163	2	1.376	0.273	0.445
SNP21	A	G	0.102	2	1.224	0.183	0.330
SNP22	T	C	0.469	2	1.993	0.498	0.691
SNP23	A	C	0.449	2	1.979	0.495	0.688
SNP24	G	A	0.102	2	1.224	0.183	0.330
SNP25	T	-	0.000	1	1.000	0.000	0.000
SNP26	C	T	0.347	2	1.829	0.453	0.646
SNP27	C	T	0.020	2	1.042	0.040	0.100

[a] Observed number of alleles. [b] Effective number of alleles. [c] Nei's gene diversity [43]. [d] Shannon's Information index.

Supplemental figures

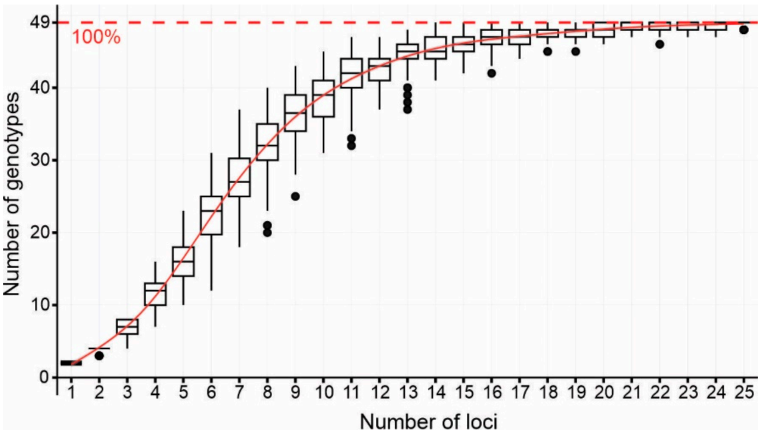

Figure S1. Number of multi-locus genotypes (MLGs) detected in *Cercospora sojina* population from Indiana, USA, by randomly sampling of a subset of 26 polymorphic SNPs markers.

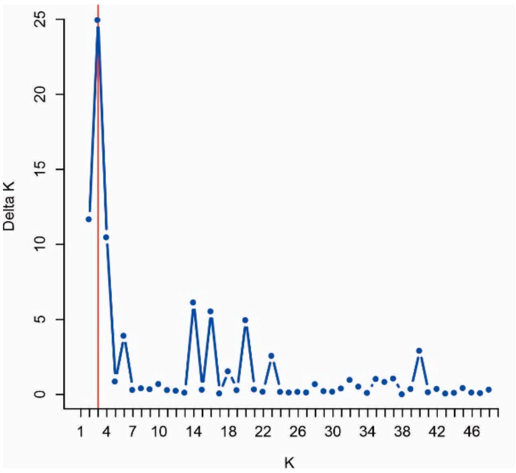

Figure S2. Delta K graph for each K cluster.
